# Strong Hsp90α/β Protein Expression in Advanced Primary CRC Indicates Short Survival and Predicts Response to the Hsp90α/β-Specific Inhibitor Pimitespib

**DOI:** 10.3390/cells14110836

**Published:** 2025-06-03

**Authors:** Sebastian B. M. Schmitz, Jakob Gülden, Marlene Niederreiter, Cassandra Eichner, Jens Werner, Barbara Mayer

**Affiliations:** 1 Department of General, Visceral and Transplant Surgery, Ludwig-Maximilians-University Munich, Marchioninistraße 15, 81377 Munich, Germany; sebastian.schmitz@med.uni-muenchen.de (S.B.M.S.); marlene.niederreiter@med.uni-muenchen.de (M.N.); cassandra.eichner@med.uni-muenchen.de (C.E.); jens.werner@med.uni-muenchen.de (J.W.); 2 German Cancer Consortium (DKTK), Partner Site Munich, Pettenkoferstraße 8a, 80336 Munich, Germany; 3 SpheroTec GmbH, Am Klopferspitz 19, 82152 Martinsried, Germany

**Keywords:** CRC, Hsp90α/β, patient stratification, prognosis, pimitespib, TAS-116, ganetespib, chemosensitization, patient-derived cancer spheroid model, personalized therapy

## Abstract

The prognosis of advanced (UICC IIb-IV) primary colorectal cancer (pCRC) remains poor. More effective targeted therapies are needed. Heat shock protein 90 alpha/beta (Hsp90α/β) expression was immunohistologically quantified in 89 pCRCs and multivariately correlated with survival. Pimitespib (Pim, TAS-116), a Hsp90α/β-specific inhibitor, was tested in pCRC cell lines and patient-derived cancer spheroids (PDCS) and referenced to the pan-Hsp90 inhibitor ganetespib (Gan, STA-9090) and standard-of-care therapies. A total of 26.97% pCRCs showed strong tumoral Hsp90α/β expression (Hsp90α/β > 40%), which correlated with reduced PFS (HR: 3.785, 95%CI: 1.578–9.078, *p* = 0.003) and OS (HR: 3.502, 95%CI: 1.292–9.494, *p* = 0.014). Co-expression of Hsp90α/β > 40% with its clients *BRAF-V600E* and Her2/neu aggravated the prognosis (*BRAF-V600E* mutated: PFS, *p* = 0.002; OS, *p* = 0.012; Her2/neu score3: PFS, *p* = 0.029). The prognostic cut-off Hsp90α/β > 40% was also a predictor for response to Pim-based therapy. Pim efficacy was increased in combination with 5-FU, 5-FU + oxaliplatin, and 5-FU + irinotecan (all *p* < 0.001). Pim induced sensitization to all chemotherapies in HT-29 (*p* < 0.001), Caco-2 (*p* < 0.01), and HCT116 (*p* < 0.05) cells. Pim combined with encorafenib in HT-29 and with trastuzumab in Caco-2 cells was most effective in dual-target inhibition approaches (HT-29: *p* < 0.005; Caco-2: *p* < 0.05). The anti-cancer effect and chemosensitization of Pim-based therapy were prospectively confirmed in PDCS directly generated from Hsp90α/β > 40% pCRCs. Protein profiling combined with functional drug testing stratifies Hsp90α/β > 40% pCRC patients diagnosed with UICC IIb-IV for effective Pim-based therapy.

## 1. Introduction

A substantial number of colorectal cancer (CRC) patients diagnosed with a locally advanced disease (Union Internationale Contre le Cancer (UICC) IIb–IIIc) develop an early recurrence (<16 months after surgery, [1]). The patient subgroups with pT4-, pN-positive tumors and patients with pN2 tumors suffer an even worse prognosis, comparable with a metastatic disease [2]. In this locally advanced stage, primary CRC (pCRC) patients are treated with adjuvant chemotherapy, while targeted therapy is currently not approved. In contrast, for pCRC patients diagnosed with synchronous metastasis (UICC IV), several targeted therapies are available for first-line treatment, depending on the biology of the individual tumor [3,4,5,6]. Most targeted therapies approved for metastatic CRC share the limitation that, when given as a single agent, they only affect a relatively small fraction of suitable patients or often offer a limited benefit for survival [7]. In fact, most CRC patients with synchronous metastasis suffer recurrence despite intensive treatment [8,9]. The five-year overall survival (OS) of those patients is less than 15% [10]. This data underlines a dire need for more effective treatment options for both locally advanced and metastatic CRC.

One of the most promising anti-cancer drugs is pimitespib (Pim, TAS-116), which was approved by the Japanese FDA for the treatment of refractory gastrointestinal stromal tumors (GISTs, [11]). Pim is currently being tested on a wide spectrum of other types of cancers [12,13], demonstrating continued interest in this compound in both Eastern and Western countries. Pim specifically targets the cytosolic isoforms of the heat shock protein 90 (Hsp90) family, known as Hsp90α and Hsp90β. Both isoforms are molecular chaperones and play key roles in the maintenance of the cell proteome [14]. They are highly homologous and interact with a broad spectrum of overlapping client proteins, many of which direct oncogenic signaling pathways [15,16]. The molecular chaperon Hsp90α/β also represents an attractive therapeutic target for advanced pCRC. Cancer cells show a much higher Hsp90α/β expression compared to normal cells, especially under stress factors [17], and are therefore more susceptible to Hsp90α/β inhibition [18].

Similarly, benign colorectal mucosa showed significantly lower Hsp90 mRNA and protein expression levels compared to the corresponding pCRC samples [19,20]. In normal mucosa, the cytosolic isoform Hsp90ß is constitutively expressed in the crypts and regulates the protein folding, maturation, stabilization, and trafficking of a variety of proteins under physiological and stress conditions. Besides the Hsp90ß isoform, precancerous mucosa and dysplastic adenomas also showed an upregulation of the inducible Hsp90α isoform. This suggests the involvement of both isoforms, Hsp90α/ß, in colorectal tumorigenesis [21]. Heterogeneous expression of Hsp90α/ß was found in pCRC, with the strongest Hsp90α/ß detection in CRC with poorly differentiated histology, an invasive phenotype, and metastatic lesions [21]. Hsp90α/ß expression was upregulated in growing tumors in response to stress conditions such as changes in the microenvironment and oncogenic pressure, suggesting that Hsp90α/ß is critical for invasion and metastasis. Indeed, Hsp90α/ß controls a number of critical signaling pathways involved in angiogenesis, epithelial–mesenchymal transformation, apoptosis resistance, metastasis, and drug resistance [22], which, taken together, lead to poor prognosis in CRC [20,23].

Several strategies are conceivable as to how Pim can expand the existing therapeutic concepts for CRC: firstly, Pim could be used as a single agent. It binds itself to the ATP-binding pocket of the N-terminal domain and the hydrophobic sub-pocket, directly inhibiting the Hsp90α and Hsp90ß isoforms [24]. Secondly, in combination with standard chemotherapeutics, Pim may improve response to chemotherapy by inhibiting Hsp90α/β client proteins that mediate chemoresistance, for example, thymidylate synthase against 5-FU [25] and XRCC1 Arg399Gln or DNA polymerases ŋ against oxaliplatin [26]. Thirdly, using the concept of dual-target inhibition, Pim could be combined with guideline-recommended drugs targeting CRC-driving client-proteins of Hsp90α/β. Examples include cetuximab directed against EGF-R, trastuzumab plus lapatinib both directed against Her2/neu, cetuximab plus encorafenib, which is directed against *BRAF V600E*, and Pembrolizumab directed against PD-L1. In fact, several clinical trials in advanced cancer patients combine Pim with a second targeted therapy, for example, with imatinib (targeting multiple tyrosine kinases in GIST, NCT05245968) [27], zimberelimab (a PD-1 inhibitor in PDAC, CRC and NSCLC, NCT04999761) [28], enzalutamide (antagonizing the androgen receptor in prostate cancer, jRCT2031230263) [29], and niraparib (a Poly(ADP-ribose) Polymerase (PARP) inhibitor in solid tumors, jRCT2031220179) [30]. This leads to the hypothesis that Pim-based therapy could represent a new treatment option for advanced pCRC, given that CRC patients can be stratified accordingly.

There is currently hardly any experimental [31,32] or clinical data [33,34] on Pim in CRC. The only study that included a larger number of CRC patients combined Pim with the checkpoint inhibitor nivolumab and showed an anti-tumor effect with manageable side effects [33]. It should be noted that in none of the introduced clinical studies, patients were biomarker–stratified for Pim treatment.

The present study aims to achieve two goals: firstly, to demonstrate that Hsp90α/β protein expression is suitable as a stratification marker for Pim-based therapy; and secondly, in a translational aspect, to determine which treatment setting shows the highest treatment efficacy of Pim therapy in advanced pCRC. A quantitative threshold of tumoral Hsp90α/β protein expression was identified that predicts both reduced survival and improved response to the Hsp90α/β-specific inhibitor Pim. The inclusion of Pim could expand the repertoire of treatment options for advanced pCRC. The introduction of a Hsp90α/β-related stratification marker could improve future trial design and identify the most suitable CRC patients for Hsp90α/β inhibitor therapy.

## 2. Patients and Methods

### 2.1. Study Population

Tumor samples from 89 patients with primary colorectal cancer (pCRC) were included.

Tumor samples were provided by the Biobank of the Department of General, Visceral and Transplantation Surgery at Ludwig Maximilians University (LMU) under the administration of the Human Tissue and Cell Research (HTCR) Foundation. The framework of the HTCR Foundation, which includes obtaining written informed consent from all donors, has been approved by the ethics commissions of the Faculty of Medicine at the University Regensburg (approval number 99/46, 31 January 2002) and of the Faculty of Medicine at the LMU (approval number 025-12, 14.01.2014) in Germany. Tumor samples were also provided by five colorectal cancer (CRC) centers—the University Hospital of LMU Munich; the Klinikum Rechts der Isar, Technical University of Munich; the Klinikum Landshut; the Klinikum Kaufbeuren; and the Klinikum Neuperlach—as part of the SpheroPCT study approved by the ethics commission of the Faculty of Medicine at LMU (approval number 252/04, 20 June 2011). Written informed consent was obtained from all patients.

All samples were received between November 2003 and September 2011. Follow-up was carried out until December 2023. The clinical and pathological parameters, which were determined according to the international guidelines for CRC from 2003 [35] and 2011 [36], were provided by the cancer centers or the biobank. At that time, the molecular pathological evaluation of *KRAS*, *BRAF*, and Her2/neu was not yet routine and was therefore analyzed in the present study. Patients were excluded if they passed away within 30 days after surgery, suffered from another neoplasia within the last five years, or developed tumors due to a hereditary disposition for CRC.

Two patients had to be excluded from survival analysis: one patient developed an additional carcinoma, while another patient lacked sufficient follow-up data. The terms sex, male, and female are used throughout the study to indicate the biological sex of the patients.

### 2.2. Tumor Sample Preparation

After surgical removal, a portion of each fresh tumor sample was immediately snap-frozen in liquid nitrogen for immunohistochemistry and molecular profiling. For 3D modeling, vital tumor tissue obtained from four additional pCRC patients was used to generate patient-derived cancer spheroids (PDCS). Snap-frozen tumors were embedded in Tissue-Tec O.C.T.^TM^ (Sakura Finetek Europe, Leiden, NL, USA; SA62550-01). Serial frozen sections (5 µm) were prepared using the CM 1950 cryostat microtome (Leica Biosystems, Nussloch, Germany). Standard hematoxylin and eosin (H&E) staining was performed to assess the quality of the sections, the tumor morphology, and the fraction of tumor cells representative of the characteristics reported by the pathologist.

### 2.3. Immunohistochemistry and Evaluation of Biomarker Expression

Protein expression was analyzed on frozen tumor sections (5 µm) using the standard avidin–biotin–peroxidase complex method as described before [37]. Tumor sections were fixed in acetone for eight minutes. Unspecific Fc-receptors were blocked with 10% AB-Serum (Bio-Rad, Hercules, CA, USA; 805135) for 20 min. Endogenous biotin was blocked for 15 min using the Avidin–Biotin Blocking Kit (Vector laboratories, Burlingame, CA, USA). Primary antibodies were applied for one hour. This included the monoclonal antibody (mab) AC88, which specifically recognizes the Hsp90α/β protein [38]; the mab 4B5, which is approved for the staining of the Her2/neu antigen [39]; as well as the anti-EpCAM mab; BerEP4 and the anti-pan cytokeratin mab AE1/AE3, which both detect epithelial cancer cells. The corresponding isotype controls, i.e., the mabs MOPC-21 and DA1E, were also considered. The primary antibodies were identified with the species-specific biotin–avidin detection system. All details about the primary and secondary antibodies and detection systems are given in Appendix A.1. The antigen–antibody reaction was visualized by incubating the sections in a 3-Amino-9-ethylcarbazol (Sigma-Aldrich, St. Louis, MO, USA, Cat No 132-32-1) peroxide solution for 10 min. All sections were counterstained with Mayer’s hemalaun solution (Merck, Darmstadt, Germany; Cat No 109249) and embedded with Aquatex^®^ (Merck; Cat No 108562). The slides were evaluated semiquantitatively using light microscopy. The percentage of positively stained carcinoma cells is given for each antigen. Her2/neu expression was analyzed according to the score established in gastric cancer [40].

### 2.4. Molecular Pathology

All primary tumor samples were characterized for *KRAS* and *BRAF V600E* status using two different pyrosequencing techniques [41,42]. Cryo-sections were micro-dissected to enrich tumor cell population. Whole genomic DNA was extracted from micro-dissected tumor areas using the QIAamp DNA Mini Kit (Qiagen, Hilden, Germany; 51304) according to the manufacturer’s instructions. *KRAS* codons 12 and 13 and *BRAF V600E* mutation sites were amplified by a polymerase chain reaction (PCR). Primer sequences, manufactured by Metabion International AG (Planegg, Germany), are shown in Appendix A.2. Automated sequencing was performed with PyroGold Q24 reagents (Qiagen; Cat No 970802) on a PyroMark Q24 instrument (Qiagen) at room temperature according to the manufacturer’s manual. Positive and negative controls were included in the analyses.

### 2.5. The Colorectal Patient-Derived Cancer Spheroid Model

Patient-derived cancer spheroids (PDCS) were directly prepared from fresh pCRC samples using a modified liquid overlay technique as described before [43]. In short, a single-cell suspension was prepared from the individual tumor sample by mechanic–enzymatic digestion using the Liberase enzyme mix according to the instructions of the manufacturer (Roche, Mannheim, Germany, Cat No 05401127001). Cell viability was determined using the trypanblue exclusion assay, and 50,000 viable cells were seeded per well in a 96-well plate. A single spheroid was generated in each well. After 48 h of formation, the spheroids were used for treatment experiments.

### 2.6. Cell Line Culture

The pCRC cell lines HCT 116 (ATCC, Manassas VA, USA, CCL-247), HT-29 (ATCC, HTB-38), Caco-2 (ATCC, HTB-37), DLD-1 (ATCC, CCL-221), LS 174T (ATCC, CL-188), and SW1116 (Merck, 87071006-1VL) were cultured in RPMI1640, containing L-Glutamine, (GIBCO, ThermoFisher, Waltham, MA, USA; Cat No 11875085) supplemented with 10% FCS (CORNING, Corning, NY, USA, Cat No 35-079-CV). Cells were kept at 37 °C and 5% CO_2_. The detachment of the cells from the culture vessels was performed with Accutase (Biolegend, San Diego, CA, USA; Cat No 423201) to preserve biomarker protein expression.

### 2.7. Cytospin Preparation

For cytospin preparation, single-cell suspensions were prepared from the pCRC cell lines. After detachment, the cells were washed twice using D-PBS (Pan Biotech, Aidenbach, Germany; Cat No P04-35500) supplemented with 10% FCS. Cytospins were prepared by spinning 50,000 cells on superfrost plus slides (Thermofisher, Waltham, MA USA; Cat No S8902) using a cytospin 3 centrifuge (Thermofisher, Waltham, MA, USA) according to the manufacturer’s instructions. After centrifugation, the cells were fixated in acetone for 10 min at −20 °C, airdried overnight, and kept at −80 °C until used for immunocytochemistry.

### 2.8. Treatment and Evaluation

For treatment of the pCRC cell lines, a single-cell suspension of each cell line was seeded in a 96-well plate at a density of 10,000 vital cells per well. After adherence, 2D cell cultures were treated with the Hsp90α/β-specific inhibitor Pim at a clinically relevant peak plasma concentration (PPC; 1.625 µg/mL, 3.58 µM). The well-known pan-Hsp90 inhibitor Gan was used as a benchmark at a PPC of 4.41 µg/mL (12.10 µM). Chemosensitization analyses were performed with the standard chemotherapies for CRC 5-fluorouracil (F), F + oxaliplatin (FO), and F + irinotecan (FI). Dual-target inhibition experiments were performed with the BRAF V600 inhibitor encorafenib and the Her2/neu inhibitor trastuzumab. Corresponding solvent controls (SCs) were considered in each treatment experiment. Further details about the drugs are given in Appendix A.3. The cell viability of the 2D-cultures was measured 24, 48, and 72 h after treatment using the CellTiter-Glo^®^Luminescent Cell Viability Assay (Promega, Fitchburg, WI, USA; Cat No G7573) according to the manufacturer’s protocol with the FilterMax F3 reader (Molecular Devices, San Jose, CA, USA). Treatment efficacy was calculated as the percentage of the mean residual metabolic activity in relation to the SC. PDCS therapy was performed for 72 h as reported previously [43], according to the same treatment plan as described above for the 2D-cultures. One representative experiment is visualized.

### 2.9. Statistical Analysis

Hsp90α/β protein expression was correlated with clinical–pathological factors using Fisher’s exact two-tailed test, including Bonferroni adjustment and the corresponding odds ratio. Overall survival (OS) was defined as the time from surgical intervention to the time of tumor-related death or last follow-up. Progression-free survival (PFS) was defined as the time between surgery and the diagnosis of the first recurrence of any kind or until the last follow-up. Patients who died of another cause were censored at their date of death. OS and PFS were estimated using the Kaplan–Meier analysis (log-rank test) including the risk ratio. The multivariate Cox proportional hazard regression model was calculated. Data from cell culture experiments was assessed using a one-way ANOVA analysis including the Tukey or Games–Howell post hoc tests. Combination therapies were evaluated for synergistic and additive effects using the combination subthresholding approach [44]. *p*-values < 0.05 were considered to be significant, while *p*-values between 0.05 and 0.10 were considered to be marginally significant.

Statistical analyses were performed using SPSS 29 (version 29.0.0.0, IBM, Armonk, NY, USA). Visualization was performed using GraphPad Prism 9 (version 9.1.2, GraphPad Software, Boston, MA, USA).

## 3. Results

### 3.1. Patient Characteristics and Survival

Patient characteristics were available for 89 patients, and follow-up data (median: 75.66 months, range 2.20 to 209.22 months) was available for 87 patients (Table 1). The median age of the patients was 69 years (mean 67.51 years, range 43–90 years). Most tumors were classified as adenocarcinomas (85.39%). A substantial number of the tumors showed an undifferentiated histology (G3/G4, 36.0%). Many patients (47.19%) were diagnosed in an advanced tumor stage (UICC IIB-IV). The significant univariate survival factors—UICC stage, grading, and resection status—which were the only variables available for all 87 patients, were included in the multivariate Cox regression model.

### 3.2. Hsp90α/β Protein Expression in Primary CRC and Survival

**Figure 1:**
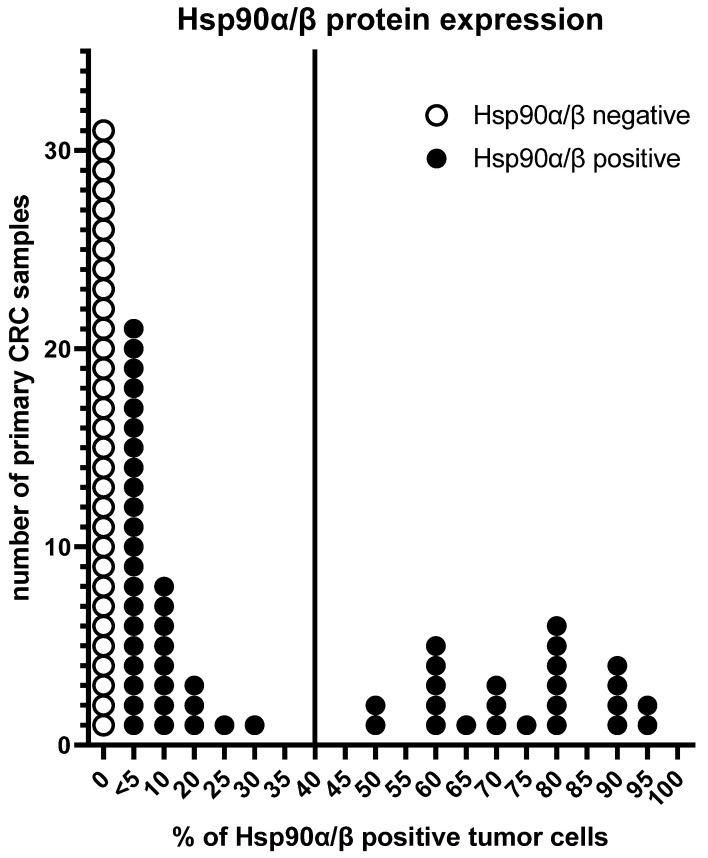
Heterogeneity of Hsp90α/β protein expression in pCRC. The vertical line indicates the cut-off ≤ 40% vs. >40% Hsp90α/β-positive tumor cells according to its biphasic distribution.

Hsp90α/β protein expression in pCRC was heterogeneous, ranging from few (<5%) to almost all (95%) Hsp90α/β-positive tumor cells. A biphasic distribution of the Hsp90α/β-positive tumor cells was observed. Therefore, the cut-off for strong Hsp90α/β protein expression was defined as >40% Hsp90α/β-positive cancer cells. Strong Hsp90α/β protein expression was detected in 26.97% (24/89) of pCRCs (Figure 1).

**Figure 2:**
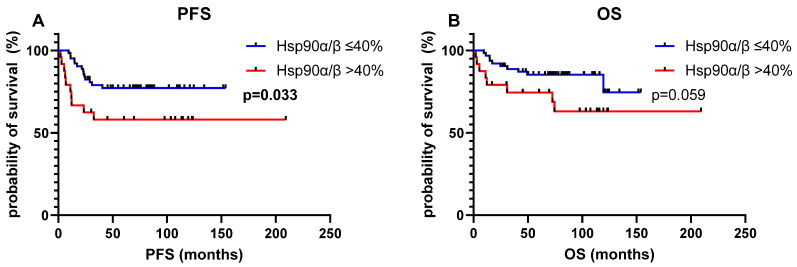
Effect of the cut-off of Hsp90α/β protein expression (≤40% vs. >40% Hsp90α/β-positive tumor cells) on survival. Kaplan–Meier survival curves, log-rank test. (**A**) Progression-free survival (PFS); (**B**) Overall survival (OS).

CRC patients with a strong Hsp90α/β-positive primary tumor showed shorter progression-free survival (PFS, *p* = 0.033, Figure 2A) and reduced overall survival (OS, *p* = 0.059, Figure 2B). Multivariate Cox regression analysis confirmed a strong Hsp90α/β protein expression as an independent factor of poor outcome (PFS: HR 3.785, 95%CI: 1.578–9.078, *p* = 0.003 and OS: HR 3.502, 95%CI: 1.292–9.494, *p* = 0.014, Table 2).

No correlation was observed between strong Hsp90α/β protein expression and other indicators of poor outcome (Table S1).

The UICC subgroup analysis revealed that the strong Hsp90α/β protein expression impaired survival in locally advanced (UICC IIB-IIIc, PFS: *p* = 0.006; OS: *p* = 0.037) and metastatic (UICC IV, PFS/OS: *p* = 0.002) but not locally restricted pCRC (UICC I-IIa, Figure S1).

**Figure 3:**
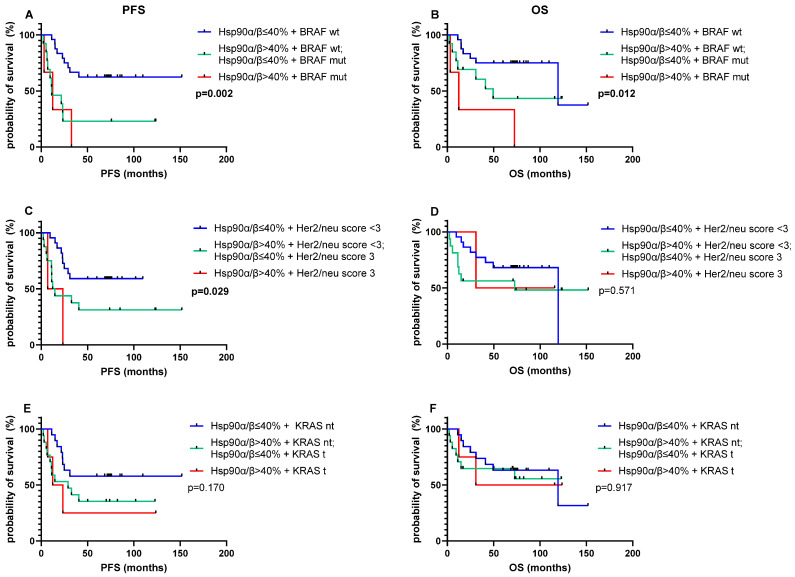
Effect of dual biomarker expression on survival of pCRC. PFS (**A**,**C**,**E**) and OS (**B**,**D**,**F**). Kaplan–Meier survival curves and log-rank test. Tumorous Hsp90α/β expression and BRAF (**A**,**B**), Her2/neu (**C**,**D**), and *KRAS* (**E**,**F**). *KRAS* t, *KRAS* treatable, includes all left-sided *KRAS* wt, G13D and G12C mut tumors; *KRAS* nt, *KRAS* non-treatable, includes all right-sided tumors and left-sided tumors with *KRAS* mut other than *KRAS* G13D and G12C; *BRAF*, *BRAF V600E*; mut, mutated; wt, wildtype.

Combinatorial biomarker analysis was performed to specify the prognosis. Hsp90α/β status was combined with *KRAS*, *BRAF V600E*, and Her2/neu status, all of which are prognostic and predictive factors in advanced pCRC. Dual biomarker expression of strong Hsp90α/β protein expression and *BRAF-V600E* mutation (3/40, 7.50%) impaired prognosis. Patients with dual biomarker expression had a shorter median PFS of 16.04 months, in comparison to patients with mixed-type tumors, which express only one of the two poor prognostic factors (13/40 (32.50%), median PFS 37.84 months) and Hsp90α/β ≤ 40% + /*BRAF V600E* wt tumors (24/40 (60.00%), median PFS 103.49 months, *p* = 0.002). Similarly, the median OS was also significantly reduced (Hsp90α/β > 40% + /*BRAF V600E* mut, median OS 29.35 months; mixed-type tumors, median OS 66.22 months; Hsp90α/β ≤ 40% + /*BRAF V600E* wt, median OS 106.50 months, *p* = 0.012). The protein co-expression of Hsp90α/β > 40%+ and Her2/neu Score 3 (2/40, 5.00%) also worsened the median PFS, which was 15.19 months in contrast to the mixed-type tumors (16/40 (40.00%), median PFS 56.88 months), and the Hsp90α/β ≤ 40% + /Her2/neu Score <3 (22/40 (55.00%), median PFS 73.67 months, *p* = 0.029). The combination of Hsp90α/β status with *KRAS* status had no effect on survival (Figure 3).

### 3.3. Colorectal Cancer Cell Line Characteristics

For Hsp90 inhibitor studies, a panel (n = 6) of well-defined pCRC cell lines was tested for Hsp90α/β protein expression. All pCRC cell lines showed a strong Hsp90α/β protein expression above the prognostic cut-off, i.e., >40% Hsp90α/β-positive cancer cells, which was independent from the clinical–pathological characteristics of the cell lines (Table 3). Caco-2 cells, which also strongly express Her2/neu protein, and HT-29 cells, which have a *BRAF V600E* mutation, were used for dual-target inhibition.

### 3.4. Hsp90 Inhibition in Primary Colorectal Cancer Cell Lines

#### 3.4.1. Hsp90 Inhibitors as Single Agents

**Figure 4:**
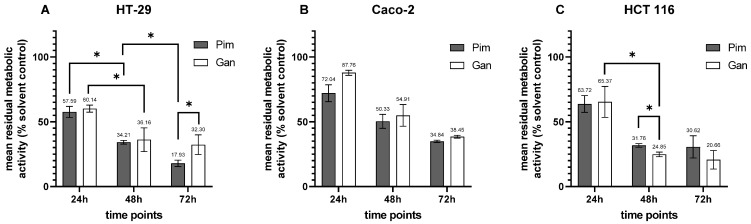
Treatment of pCRC cell lines with Hsp90 inhibitors as single agents for 24 h, 48 h, and 72 h. (**A**) HT-29; (**B**) Caco-2; (**C**) HCT 116. Pim, pimitespib; Gan, ganetespib, * *p* < 0.05.

Hsp90 inhibitors tested as single agents elicited different responses depending on the cancer cell line and the duration of treatment. HT-29 cells were more sensitive to Pim (Pim vs. Gan, *p* = 0.039 at 72 h), and effectiveness increased over the course of treatment with both Pim (24 h vs. 48 h, *p* = 0.003; 48 h vs. 72 h, *p* = 0.018) and Gan (24 h vs. 48 h, *p* = 0.010, Figure 4A). In comparison to HT-29, Caco-2 cells were less sensitive to both Hsp90 inhibitors at all treatment time points (Figure 4B). HCT 116 cells were more susceptible to Gan (Gan vs. Pim, *p* = 0.049, at 48 h), and Gan showed an increasing effect over treatment time (24 h vs. 48 h, *p* = 0.001, Figure 4C). None of the other comparisons between the Hsp90 inhibitors and the treatment time points were significant.

#### 3.4.2. Hsp90 Inhibitors in Combination Therapy

**Figure 5:**
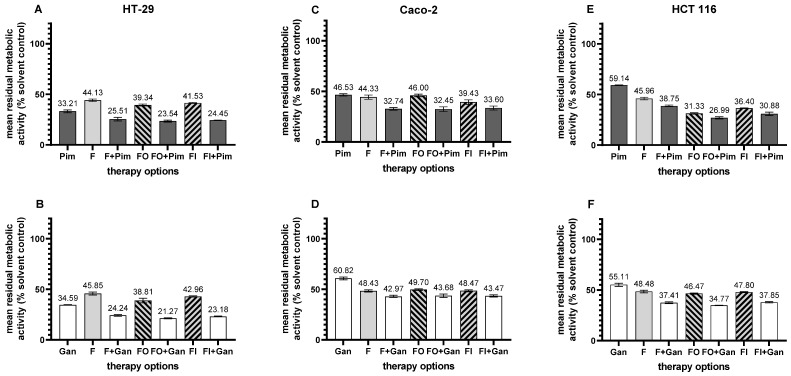
Treatment of pCRC cell lines with Hsp90 inhibitors in combination with standard chemotherapy. (**A**,**B**) HT-29; (**C**,**D**) Caco-2; (**E**,**F**) HCT 116. (**A**,**C**,**E**) pimitespib (Pim)-related therapy; (**B**,**D**,**F**) ganetespib (Gan)-related therapy. F, 5-fluorouracil; FO, 5-fluorouracil + oxaliplatin; FI, 5-fluorouracil + irinotecan. Statistical data is presented in Table S2.

The combination of Pim with guideline-recommended chemotherapy resulted in an increased anti-cancer effect compared to either agent alone. Pim efficacy was increased in combination with 5-fluorouracil (F), F + oxaliplatin (FO), and F + irinotecan (FI) in all three tested pCRC cell lines (all *p* < 0.001). In HT-29 cells, chemosensitization induced by Pim was observed in combination therapy with F, FO, and FI (F vs. F + Pim, FO vs. FO + Pim, FI vs. FI + Pim, all *p* < 0.001, Figure 5A). Similarly, an increase in anti-cancer activity was found in combining Gan with standard chemotherapy (F vs. F + Gan, *p* < 0.001; FO vs. FO + Gan, *p* = 0.002; FI vs. FI + Gan, *p* < 0.001, Figure 5B). Interestingly, the effect of F combined with an Hsp90 inhibitor was superior to a standard doublet (F + Pim vs. FO, *p* < 0.001, F + Pim vs. FI, *p* < 0.001, F + Gan vs. FO, *p* = 0.003, F + Gan vs. FI, *p* < 0.001). The chemosensitizing effect induced by Hsp90 inhibitors was further confirmed in Caco-2 cells (Figure 5C,D) and HCT 116 cells (Figure 5E,F). All combinations of Hsp90 inhibitors with standard chemotherapies achieved a significantly increased anti-cancer effect, which is documented in detail in Table S2. All Hsp90 inhibitor-based combination therapies were considered synergistic according to the combination subthresholding analysis.

**Figure 6:**
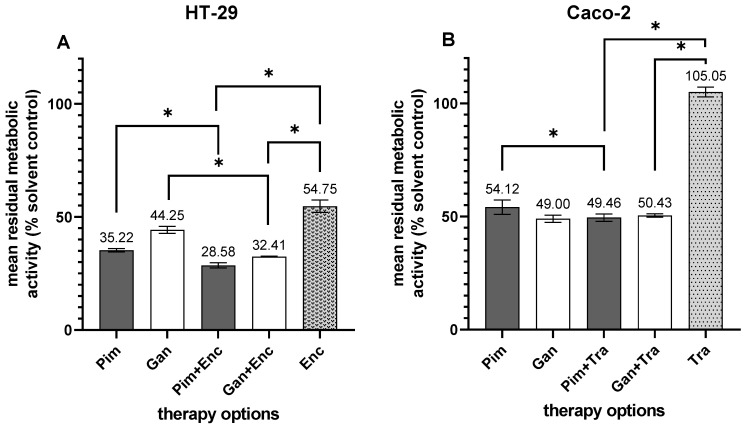
Treatment of pCRC cell lines with Hsp90 inhibitors in combination with standard targeted therapy. (**A**) HT-29; (**B**) Caco-2; Pim, pimitespib; Gan, ganetespib; Enc, encorafenib; Tra, trastuzumab, * *p* < 0.05.

In addition, Hsp90 inhibitors were combined with targeted therapy approved for metastatic CRC. Both Pim and Gan combined with the BRAF inhibitor encorafenib (Enc) were more effective against *BRAF V600E*-mutated HT-29 cells compared to each drug alone (Pim vs. Pim + Enc, *p* = 0.001; Gan vs. Gan + Enc, *p* = 0.002; Enc vs. Pim + Enc, *p* < 0.001; Enc vs. Gan + Enc, *p* = 0.002; Figure 6A). Caco-2 cells overexpressing the Her2/neu antigen were most effectively inhibited by Gan as a single agent, followed by the combination therapy of trastuzumab (Tra), Pim (Pim vs. Pim + Tra, *p* = 0.037; Tra vs. Tra + Pim, *p* < 0.001), and Gan (Tra vs. Gan + Tra, *p* < 0.001; Figure 6B). Both Pim-based combination therapies were considered synergistic according to the combination subthresholding analysis.

### 3.5. Hsp90 Inhibition in the Colorectal Patient-Derived Cancer Spheroid Model

**Figure 7:**
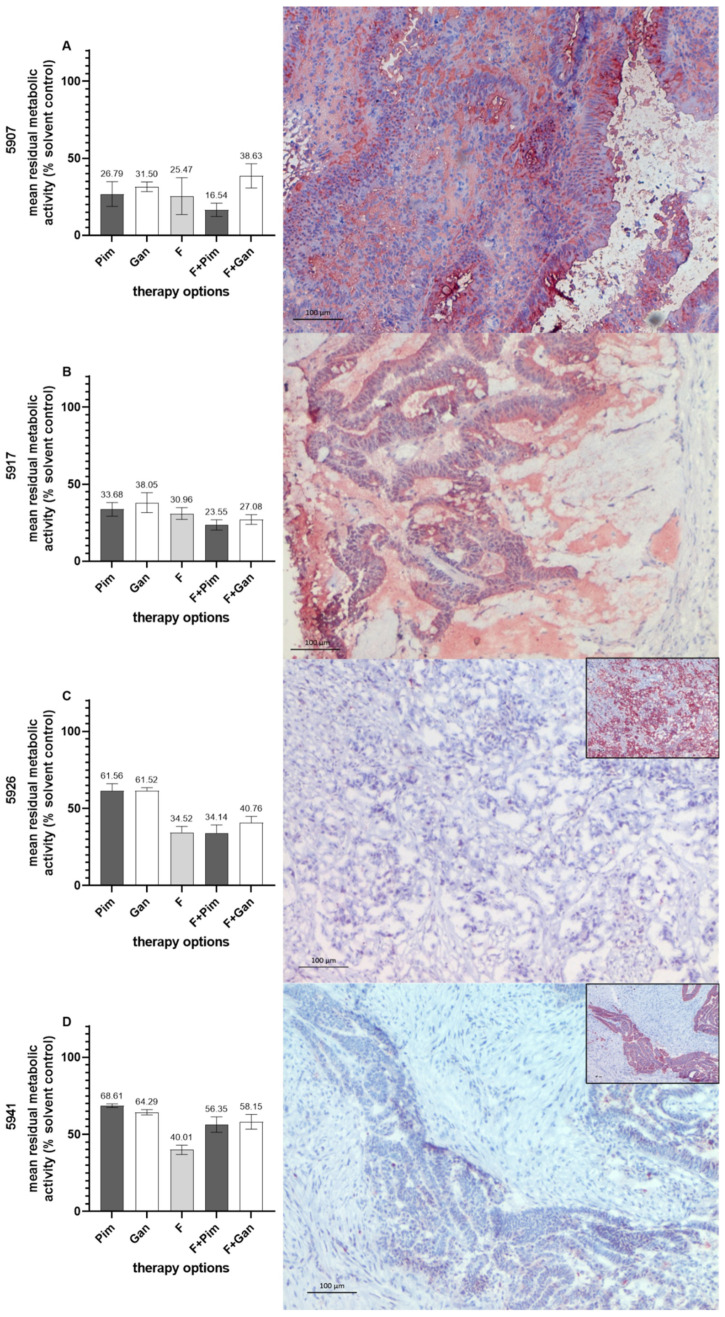
Treatment of colorectal PDCS with Hsp90 inhibitors as single agents and in combination with standard chemotherapy (left-sided panel). Hsp90α/β protein expression on tumor sections of the pCRC samples from which the PDCS were directly prepared (right-sided panel). (**A**) patient ID 5907; (**B**) patient ID 5917; (**C**) patient ID 5926; (**D**) patient ID 5941. In (**C**,**D**), the pan-cytokeratin marker AE1/AE3 as a positive control is given (small figures). Pim, pimitispib; Gan, ganetespib; F, 5-fluorouracil. Bars indicate 100 µm.

Strong Hsp90α/β protein expression (>40% Hsp90α/β-positive cancer cells) was prospectively evaluated as a predictor for response to Hsp90 inhibitors. For this purpose, patient-derived cancer spheroids (PDCS) were directly prepared from pCRC samples without knowledge of the Hsp90α/β status and treated with Hsp90 inhibitors. Treatment responses were correlated with Hsp90α/β protein expression in the primary tumor samples the spheroids were generated from. Treatment with Pim and Gan was twice as effective in PDCS directly generated from advanced pCRC samples with a strong Hsp90α/β protein expression (patient IDs 5907 and 5917). Additionally, in the PDCS models from both patients, the combination of the Hsp90 inhibitors with F resulted in chemosensitization (Figure 7A,B). In contrast, a low anti-tumor effect of Pim and Gan was observed in PDCS that were generated from advanced pCRC with a low (<40% positive tumor cells) Hsp90α/β protein expression (patient IDs 5926 and 5941). In those patients, no chemosensitization of F was observed in combination therapy with an Hsp90 inhibitor (Figure 7C,D).

## 4. Discussion

Patients diagnosed with advanced primary colorectal cancer (pCRC) have a poor prognosis and require both surgical resection and systemic therapy for first-line treatment. While chemotherapy is currently the gold standard for locally advanced pCRC, an increasing number of targeted therapies have been approved for metastatic CRC [51].

In fact, several studies have shown that Hsp90α/β is strongly expressed both at the mRNA level [52] and at the protein expression level [20,21,23] in a substantial number of pCRCs, and it is associated with tumor progression and poor prognosis. This association was confirmed in the present study, which showed that increased (>40% positive tumor cells) Hsp90α/β protein expression in locally advanced and metastatic pCRC correlated with reduced PFS and shortened OS independent of other clinicopathological prognostic factors. The biphasic Hsp90α/β status, which is a survival factor in pCRC, was also reported for distant CRC metastasis [37]. The enhanced tumorous Hsp90α/β expression promotes the activation of numerous cancer-critical signal pathways in CRC, which results in tumor growth, modulation of the tumor microenvironment, invasion, metastasis, and drug resistance (e.g., [22,53,54]). Even worse, the present study found that elevated Hsp90α/β protein levels, which are co-expressed with its client molecules and tumor drivers *BRAF V600E* mutated and Her2/neu overexpressed, further impaired the prognosis of CRC.

These biomarker-based prognostic findings suggest new Hsp90α/β-related treatment strategies for advanced pCRC. The only Hsp90 inhibitor registered to date is pimitespib (Pim), which selectively binds to both cytosolic isoforms Hsp90α and Hsp90β. However, Pim has so far only been approved by the Japanese FDA and is restricted to refractory GISTs. Interestingly, since the clinical breakthrough of Pim [11], a variety of new specific Hsp90 inhibitors [55,56,57,58] and new trial designs for Hsp90 inhibitors [59] are under development. In contrast to pan-Hsp90 inhibitors, little information is currently available for Pim in both experimental [31,32] and clinical CRC studies [33,34]. Therefore, in the present study, the anti-tumor activity of Pim was compared to that of Gan in two different in vitro models, namely the 2D pCRC cell line model and the 3D patient-derived cancer spheroid (PDCS) model. Colorectal PDCS recapitulate important biological features of the cancer cells and the tumor microenvironment [43]. Ganetespib (Gan) is one of the best-characterized pan-Hsp90 inhibitors in cancer [60], including experimental [32,61,62,63] and clinical CRC studies [64,65,66]. Unlike Pim, Gan was not approved due to an unfavorable assessment between anti-tumor efficacy and toxicity [60].

Pim as a single agent had a moderate anti-cancer effect, similar to the one observed for Gan monotherapy [62]. In all 2D and 3D cancer models with a strong (>40% positive tumor cells) Hsp90α/β protein expression, Pim qualified as a chemosensitizer potentiating the anti-tumor activity of standard-of-care chemotherapeutics. Similar to the findings in the present study, the chemosensitizing activity of Gan has been published for several CRC cell lines, and various mechanisms of action have been identified: destabilization of the Hsp90 interaction with several client proteins resulted in the downregulation of the thymidylate synthase, which is the target of 5-FU, as well as the inhibition of various proliferation signaling pathways [62,63].

Dual-target inhibition is an approved therapeutic strategy in metastatic CRC [5,6]. The combinations of Pim with the BRAF V600 inhibitor encorafenib, or with trastuzumab, which is approved for Her2/neu-positive CRC, were more effective than either drug alone. Similar results have been reported for SW480 cells using a simultaneous inhibition of Hsp90α/β and CDK4/6 with a combination of Pim plus palbociclip or abemaciclib [32]. Dual-target inhibition may represent a promising treatment strategy for CRC patient subgroups with Hsp90α/β > 40%/*BRAF V600E* mutations and Hsp90α/β > 40%/Her2neu Score 3 tumors, which both contribute to particularly poor survival. In fact, clinical activity of combined pan-Hsp90 and *BRAF V600E* inhibition was found in patients with unresectable *BRAF V600E*-mutant melanoma [67]. Similarly, the combination of a pan-Hsp90 inhibitor with an anti-Her2/neu antibody was active in the clinical setting of Her2/neu-positive metastatic breast cancer [68]. Furthermore, objective tumor response with manageable side effects was observed in advanced CRC patients treated with Pim plus the checkpoint inhibitor nivolumab [33]. These results suggest that Pim is a promising candidate for the treatment of advanced pCRCs, especially in combination with chemotherapeutics, targeted drugs, and checkpoint inhibitors, but this needs to be further investigated.

In addition to its prognostic value, the same Hsp90α/β cut-off was found to be predictive of the response to Pim. The treatment results of Pim obtained in the colorectal PDCS model were correlated with the Hsp90α/β protein expression level in the corresponding pCRC, respectively. This prospective approach suggests a strong Hsp90α/β protein expression as a stratification marker to select the CRC patients most likely to respond to Pim-based therapy. Remarkably, previous pan-Hsp90 inhibitor trials targeting advanced CRC were performed unstratified (tanespimycin [69,70], ganetespib (STA-9090) [64,65,66], luminespib (AUY922) [71,72], and onalespip [73,74]). The limited efficacy and intolerable toxicities reported in these studies emphasize the urgent need for stratifying biomarkers for the upfront selection of CRC patients who could potentially benefit from Hsp90 inhibitor therapy. Several factors were proposed for stratification. These include the consensus molecular subtypes (CMSs), the involvement of CRC-driving Hsp90 client proteins, a favorable epichaperome [25], and tumoral Hsp90 overexpression [75,76]. The cut-off value of >40% tumor Hsp90α/β protein expression determined in the present study could be an indicator for the treatment efficacy of Pim-based therapy. However, the methods presented in this study are not suitable for evaluating or predicting Pim-induced toxicities, frequently described in clinical trials, such as diarrhea, nausea, or eye disorder [11,77]. The increased expression of the Hsp90α/β protein was found in the primary tumor of a substantial subset (26.97%) of CRC patients. This prevalence is lower than that of *KRAS* treatable CRC, but higher than that of numerous drug targets approved, such as MSI-high/dMMR, NTRK fusions, Her2/neu overexpression, and *BRAF V600E* [7,78]. This finding suggests that a significant number of CRC patients would benefit from a Hsp90α/β-directed Pim-based therapy. In addition to the Hsp90α/β phenotype, the present study identified the PDCS model as an option for selecting the most effective Pim-related therapy. This functional approach offers the possibility of testing Pim as a single agent, as well as in combination with standard chemotherapeutics, small molecules, and biologicals, aiming to select the most effective Pim-based treatment in the context of a personalized therapy [79,80,81].

However, further analysis in a large patient cohort is required to confirm the proposed threshold of Hsp90α/β protein expression as a biomarker of poor prognosis and response to Pim therapy. In addition, comprehensive drug testing in the patient-derived spheroid model needs to be increased to identify those CRC patients who would benefit most from Hsp90-specific inhibitor therapies.

## 5. Conclusions

**Figure 8:**
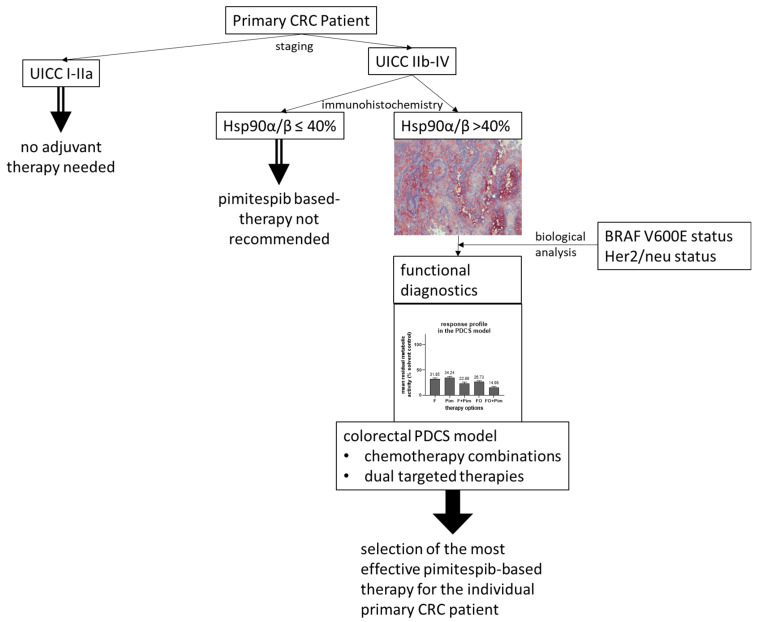
Suggested sequential diagnostic platform to stratify appropriate CRC patients for pimitespib-based therapy.

The present study supports pimitespib (Pim) as a new targeted therapy for CRC patients diagnosed with an advanced primary tumor strongly expressing the Hsp90α/β protein. Increased Hsp90α/β protein expression in primary CRC (pCRC) is an independent factor for poor prognosis in a substantial number of patients in a locally advanced and metastatic stage (UICC IIB-IV). Strong Hsp90α/β protein expression in primary tumors, in addition, might be a predictive factor for response to Pim therapy. Thus, strong Hsp90α/β protein expression in advanced pCRC could develop into a biomarker, which might enable the stratification of suitable CRC patients for Pim therapy in clinical trials. Pim as a single agent had a moderate anti-tumor effect, but it was more successful in combination with standard treatment, which resulted in chemosensitization and dual-target inhibition. The present study suggests a sequential diagnostic platform, which combines protein profiling and functional drug testing in the patient-derived cancer spheroid (PDCS) model, as a strategy to identify the most effective Pim treatment for the individual CRC patient (Figure 8).

## Figures and Tables

**Table 1:** Patient clinical–pathological characteristics and Kaplan–Meier analysis for progression-free survival (PFS) and overall survival (OS).

Factor	Groups	Frequency			PFS			OS	
		n	Distribution	Percent	n	Log-Rank, *p*-Value	Risk Ratio	Log-Rank, *p*-Value	Risk Ratio
sex		89			87				
	male		50	56.18		0.195	1.72	0.200	1.93
	female		39	43.82					
age (years)		89			87				
	median (range)		69 (43–90)						
	≤median		47	52.81		0.146	0.59	0.101	0.45
	>median		42	47.19					
tumor location		89			87				
	right-sided ^∞^		42	47.19		0.922	1.01	0.305	0.68
	left-sided #		47	52.81					
tumor diameter (cm)		89			87				
	mean (range)		4.72 (1.20–11.50)						
	≤mean		48	53.93		0.549	1.23	0.960	0.98
	>mean		41	46.07					
histological type		89			87				
	adenocarcinoma		76	85.39		0.135	1.82	0.232	1.97
	mucinous adenocarcinoma		13	14.61					
histologic grade (G)		89			87				
	G1/2		57	64.00			2.53	0.057	2.26
	G3/4		32	36.00		**0.005**			
pT stage		89			87				
	pT1/2		16	18.00			nc		nc
	pT3/4		73	82.00		**0.012**		**0.023**	
pN stage		89			87				
	pN0		57	64.00			**3.80**		**3.80**
	pN1/2		32	36.00		**<0.001**		**<0.001**	
cM stage		89			87				
	cM0		75	84.27			**6.16**		**10.43**
	cM1		14	15.73		**<0.001**		**<0.001**	
UICC		89			87				
	I-IIA		47	52.81			**12.93**		**19.98**
	IIB-IV		42	47.19		**<0.001**		**<0.001**	
lymphangiosis carcinomatosa (L) ^x^		75			73				
	L0		57	76.00			**4.80**		**6.11**
	L1		18	24.00		**<0.001**		**<0.001**	
vessel invasion (V) ^x^		76			74				
	V0		70	92.11			**3.02**		**5.04**
	V1		6	7.89		**0.001**		**<0.001**	
perineural invasion (P) ^x^		57			56				
	P0		52	91.23			**4.08**		**5.10**
	P1		5	8.77		**<0.001**		**<0.001**	
resection status (R)		89			87				
	R0		85	95.51			**4.15**		**5.93**
	R1/2		4	4.49		**<0.001**		**<0.001**	
sCEA ^x^		86			85				
	physiological		43	50.00			**3.71**		**7.81**
	pathological		43	50.00		**<0.001**		**<0.001**	

∞ right-sided includes caecum, colon ascendens, and transverse colon; # left-sided includes colon descendens, sigmoid colon, and rectum. UICC, Union Internationale contre le cancer; sCEA, soluble CEA at presurgical time point; nc, not computable; ^x^—data was not reported for all patients.

**Table 2:** Cox regression analysis for the Hsp90α/β cut-off and prognostic clinical–pathological factors.

Factor	Groups	PFS			OS		
		HR	95% CI	*p*-Value	HR	95% CI	*p*-Value
sex *	male vs. female	0.791	0.323–1.937	0.608	1.031	0.369–2.879	0.954
histological grade	G3/4 vs. G1/2	1.928	0.834–4.456	0.125	1.446	0.544–3.845	0.459
UICC stage	IIB/IV vs. I/IIA	19.667	4.194–92.235	**<0.001**	21.732	2.692–175.434	**0.004**
resection status	R1/2 vs. R0	6.886	1.957–24.223	**0.003**	12.953	3.281–51.148	**<0.001**
Hsp90α/β positive tumor cells	>40% vs. ≤40%	3.785	1.578–9.078	**0.003**	3.502	1.292–9.494	**0.014**

* sex is used as a confounder; UICC, Union Internationale Contre le Cancer; PFS, progression-free survival; OS, overall survival; HR, hazard ratio; 95% CI, 95% confidence interval.

**Table 3:** Clinical–pathological and molecular characteristics of pCRC cell lines.

	HT-29	Caco-2	HCT 116	DLD-1	LS 174T	SW1116	Source
**Biological Characteristics**							
Hsp90α/β % positive cells	90	70	95	50	95	95	Results of the present study
Her2/neu % positive cells	<10	70	<10	<10	0	0	
EpCAM % positive cells	95	95	95	95	90	95	
*BRAF V600E*	mut	wt ^$^	wt	wt	wt ^$^	wt ^$^	$ [45,46]
**Clinical–Pathological Characteristics**							
sex	female	female	male	male	female	male	[45,46,47]
age (years)	44	72	48	45	58	73	[45,46,47,48,49]
primary tumor location	colon	colon	colon ascendens	sigmoid colon	colon	colon	[45,46,47,49,50]
histological type	adeno-ca	adeno-ca	adeno-ca	adeno-ca	adeno-ca	adeno-ca	[45,47,48,49]

adeno-ca, adenocarcinoma; mut, mutated; wt, wildtype; $, data taken from references.

## Data Availability

The data presented in this study is available on request from the corresponding author.

## Supplementary Materials

The following supporting information can be downloaded at: https://www.mdpi.com/article/10.3390/cells14110836/s1, Figure S1: Correlation of the cut-off of Hsp90α/β protein expression in pCRC with different UICC-stage using Kaplan-Meier-survival analysis and the log-rank test. A, B: locally restricted (UICC I-IIa), C, D: locally advanced (UICCIIb-IIIC), E, F: metastatic (UICC IV). A, C, E: PFS, B, D, F: OS.; Table S1: Correlation of the cut-off of Hsp90α/β protein expression in pCRC with clinicalpathological factors using the Fishers’ two-tailed exact test.; Table S2: Statistical analysis of the Hsp90 inhibitors in combination with standard chemotherapy in pCRC cell lines HT-29, Caco-2 and HCT 116.

## Abbreviations

The following abbreviations are used in this manuscript:

**Abbreviation**	**Meaning**
°C	degree Celsius
µm	micro-meters
95% CI	95% confidence interval
adeno-ca	adenocarcinoma
ADP	adenosine diphosphate
ATP	adenosine triphosphate
BRAF	B-Raf proto-oncogene
*BRAF V600E*	B-Raf proto-oncogene V600E mutation
Cat No	catalog number
cM	clinical metastasis stage
CRC	colorectal cancer
dMMR	deficient mismatch repair
DMSO	dimethyl sulfoxide
DNA	desoxyribonucleic acid
D-PBS	Dulbecco’s phosphate-buffered saline
EGF-R	epithelial growth factor receptor
Enc	encorafenib
EpCam	epithelial cell adhesion molecule
F; 5-FU	5-fluorouracil
FCS	fetal cow serum
FDA	Food and Drug Administration
FI	5-fluorouracil + Irinotecan
FO	5-fluorouracil + oxaliplatin
Gan	ganetespib, STA-9090
GIST	gastrointestinal stromal tumor
H&E	hematoxylin and eosin
HER2/neu	human epidermal growth factor receptor 2
HR	hazard ratio
HRP	horseradish peroxidase
Hsp90	heat shock protein 90
Hsp90α/β	heat shock protein 90 alpha and beta isoform
HTCR	Human Tissue and Cell Research
ID	identification
*KRAS*	Kirsten rat sarcoma virus
L	lymphangiosis carcinomatosa
LMU	Ludwig Maximilians University
mab	monoclonal antibody
mRNA	messenger ribonucleic acid
MSI	microsatellite instability
mut	mutated
nc	non-computable
NSCLC	non-small-cell lung cancer
nt	non-treatable
NTRK	neurotropic tyrosinkinase
OS	overall survival
*p*	*p*-value
P	perineural invasion
PARP	poly-adenosine diphospate ribose polymerase
pCRC	primary colorectal cancer
PD-1	programmed cell death protein 1
PDAC	pancreatic ductal adenocarcinoma
PDCS	patient-derived cancer spheroid(s)
PD-L1	programmed death-ligand 1
PFS	progression-free survival
Pim	pimitespib; Tas-116
pN	pathological lymph node stage
PPC	peak plasma concentration
pT	pathological tumor stage
R	resection status
SC	solvent control(s)
sCEA	soluble carcinoembryonic antigen
t	treatable
Tra	trastuzumab
UICC	Union Internationale Contre le Cancer
V	vessel invasion
wt	wild type
XRCC1	X-ray repair cross complementing protein 1

## Appendix A

### Appendix A.1. Primary and Secondary Antibodies and the Detection Systems Used for Immunohistochemistry and Immunocytochemistry

**Antigen**	**Antibody Clone**	**Order Number**	**Host Species**	**Isotype**	**Working** **Concentration (µg/mL)**	**Kit**	**Supplier**
primary antibodies							
Hsp90α/β	AC88	ab13492	mouse	IgG1	10	+	Abcam Cambridge, UK
Her2/neu	4B5	05278368001	rabbit	IgG	1.5	-	Roche basis supplier Basel, Switzerland
EpCam	BerEp4	M080401-2	mouse	IgG1	2.5	-	Dako Jena, Germany
pan-cytokeratin	AE1/AE3	NBP2-29429	mouse	IgG1	0.5	+	Novus Biological Centennial, CO, USA
MOPC-21	MOPC-21	MACF1081Z	mouse	IgG1	10	+	Sigma-Aldrich, St. Louis, MO, USA
MOPC-21	MOPC-21	MACF1081Z	mouse	IgG1	2.5	-	Sigma-Aldrich, St. Louis, MO, USA
DA1E	DA1E	3900S	rabbit	IgG	1.5	-	CellSignaling, Danvers, MA, USA
secondary antibodies							
rabbit anti-mouse	polyclonal	315-065-045	rabbit	IgG	0.75		Jackson ImmunoResearch, West Grove, PA, USA
goat anti-rabbit	polyclonal	111-065-144	goat	IgG	7		Jackson ImmunoResearch
detection systems							
streptavidin–horseradish peroxidase	-	016-030-084	-	-	1		Jackson ImmunoResearch
Zytochem Plus HRP Kit	polyclonal	HRP 060	mouse, rabbit, rat	-	ready to use		Zytomed Systems GmbH, Berlin, Germany

### Appendix A.2. PCR and Sequencing Primers

*KRAS* codon 12 + 13 a	forward	5′-NNNGGCCTGCTGAAAATGACTGAA-3′
	reverse	5′-Bio-TTAGCTGTATCGTCAAGGCACTCT-3′
	sequencing	5′-TGTGGTAGTTGGAGCT-3′
*KRAS* codon 12 + 13 b	forward	5′-Bio-TGACTGAATATAAACTTGTGGTAGTTG-3′
	reverse	5′-TCGTCCACAAAATGATTCTGA-3′
	sequencing	5′-GCACTCTTGCCTACG-3′
*BRAF V600E*	forward	5′-TGAAGACCTCACAGTAAAAATAGG-3′
	reverse	5′-Bio-TCCAGACAACTGTTCAAACTGAT-3′
	sequencing	5′-GTAAAAATAGGTGATTTTGG-3′
a sequencing according to Ogino [41]; b sequencing according to Poehlmann [42].		

### Appendix A.3. Drugs Used for Treatment

**Name of the Drug**	**Target**	**Peak Plasma Concentration (µg/mL)**	**Solvent Control**	**Supplier**	**Order Number**	**Ref.**
pimitespib (Pim)	Hsp90α/β	1.625	DMSO	Selleckchem Cologne, Germany	S7716	[34]
ganetespib (Gan)	pan-Hsp90	4.41	DMSO	MedChem Express Monmouth Junction, NJ, USA	HY-15205	[64,82]
encorafenib (Enc)	*BRAF V600* mutation	1.556	DMSO	Selleckchem	S7108	[83]
trastuzumab (Tra)	Her2/neu protein	132.00	water	Pharmacy, LMU Munich, Germany	not available	[84]
5-fluorouracil (F)		100.00	water	Pharmacy, LMU	not available	[85]
oxaliplatin (O)		1.89	water	Pharmacy, LMU	not available	[86]
irinotecan (I)		2.10	water	Pharmacy, LMU	not available	[87]
