# Supplementary material for: Strong Hsp90α/β Protein Expression in Advanced Primary CRC Indicates Short Survival and Predicts Response to the Hsp90α/β-Specific Inhibitor Pimitespib
Source: Cells. 2025 Jun 3;14(11):836. doi: 10.3390/cells14110836 (PMC12154481; doi:10.3390/cells14110836)
Supplement: Supplementary file 1 [file cells-14-00836-s001.zip › cells-3645626-supplementary.pdf]

Supplementary Table S1. Correlation of the cut-off of Hsp90α/β protein expression in pCRC with clinical-pathological factors using the Fishers' two-tailed exact test

factor	groups	n	Hsp90α/β ≤ 40%	Hsp90α/β >40%	p
sex	male	89	34	16	0.242
	female		31	8	
age years (median)	≤ 69	89	34	13	1.000
	> 69		31	11	
tumor localization	right-sided	89	31	11	1.000
	left-sided		34	13	
tumor diameter cm (mean)	≤ 4.72	89	37	11	0.473
	>4.72		28	13	
histological type	adenocarcinoma	89	56	20	0.742
	mucinous adenocarcinoma		9	4	
histological grade	G1/2	89	46	11	**0.045**
	G3/4		19	13	
pT stage	pT1/2	89	11	5	0.758
	pT3/4		54	19	
pN stage	pN0	89	41	16	0.808
	pN1/2		24	8	
cM stage	cM0	89	55	20	1.000
	cM1		10	4	
UICC-stage	I-IIA	89	34	13	1.000
	IIB-IV		31	11	
lymphangiosis carcinomatosa	L0	75	43	14	0.140
	L1		10	8	
vessel invasion	V0	76	52	18	0.338
	V1		3	3	
perineural invasion	P0	57	37	15	1.000
	P1		4	1	
resection status	R0	89	62	23	1.000
	R1/2		3	1	
sCEA	physiological	86	30	13	0.810
	pathological		32	11	
KRAS*	treatable	89	24	10	0.807
	non-treatable		41	14	
BRAF V600E	wt	89	60	19	0.125
	mut		5	5	
Her2/neu score	<3	89	56	20	0.742
	3		9	4	

*KRAS treatable includes left-sided tumors with KRAS wt, G13D and G12C mut; KRAS non-treatable, includes all right-sided tumors and left-sided tumors KRAS mut other than KRAS G13D and KRAS G12C; mut, mutated; wt, wild-type.

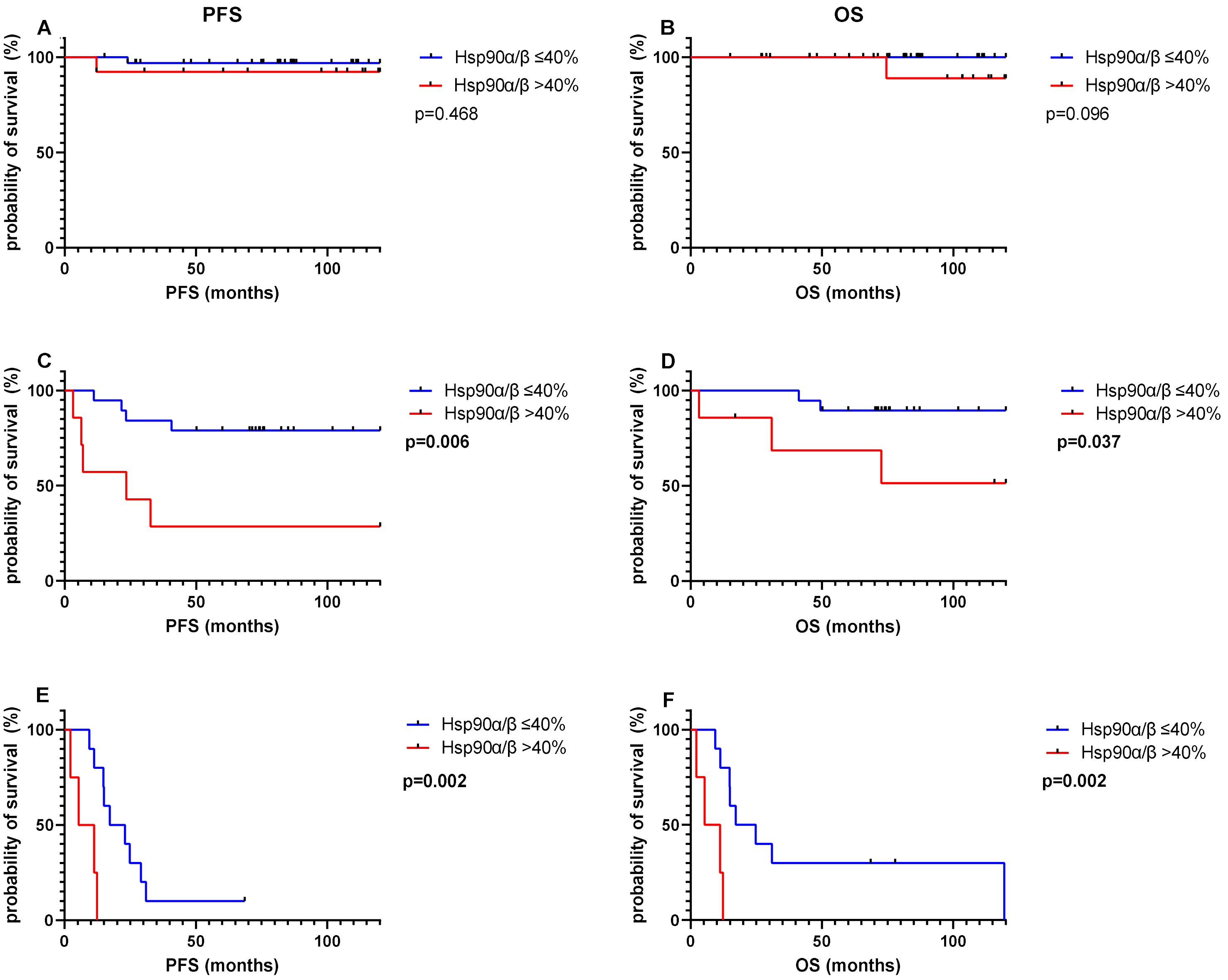

Supplementary Figure S1. Correlation of the cut-off of Hsp90α/β protein expression in pCRC with different UICC-stage using Kaplan-Meier-survival analysis and the log-rank test. A, B: locally restricted (UICC I-IIa), C, D: locally advanced (UICCIIb-IIIC), E, F: metastatic (UICC IV). A, C, E: PFS, B, D, F: OS.

Supplementary Table S2. Statistical analysis of the Hsp90 inhibitors in combination with standard chemotherapy in pCRC cell lines HT-29, Caco-2 and HCT 116

therapy 1	therapy 2	HT-29 p-value	Caco-2 p-value	favored therapy	HCT 116 p-value	favored therapy
Pim	F+Pim	**<0.001**	**<0.001**	F+Pim	**<0.001**	F+Pim

Pim	FO+Pim	**<0.001**	**<0.001**	FO+Pim	**<0.001**	FO+Pim
Pim	FI+Pim	**<0.001**	**<0.001**	FI+Pim	**<0.001**	FI+Pim
F	F+Pim	**<0.001**	**<0.001**	F+Pim	**0.002**	F+Pim
FO	FO+Pim	**<0.001**	**<0.001**	FO+Pim	**0.009**	FO+Pim
FI	FI+Pim	**<0.001**	**0.003**	FI+Pim	**0.034**	FI+Pim
F+Pim	FO	**<0.001**	**<0.001**	F+Pim	**<0.001**	FO
F+Pim	FI	**<0.001**	**<0.001**	F+Pim	**0.046**	FI
Gan	F+Gan	**<0.001**	**<0.001**	F+Gan	**<0.001**	F+Gan
Gan	FO+Gan	**<0.001**	**<0.001**	FO+Gan	**<0.001**	FO+Gan
Gan	FI+Gan	**<0.001**	**<0.001**	FI+Gan	**<0.001**	FI+Gan
F	F+Gan	**<0.001**	**<0.001**	F+Gan	**<0.001**	F+Gan
FO	FO+Gan	**0.002**	**<0.001**	FO+Gan	**<0.001**	FO+Gan
FI	FI+Gan	**<0.001**	**<0.001**	FI+Gan	**<0.001**	FI+Gan
F+Gan	FO	**0.003**	**<0.001**	F+Gan	**<0.001**	F+Gan
F+Gan	FI	**<0.001**	**<0.001**	F+Gan	**<0.001**	F+Gan
